# New Orleans

**Published:** 1854-07

**Authors:** 


*New Orleans.*

New Orleans, during the past season, presented a spectacle not often heretofore witnessed on any part of our earth. Diseases are something with which we have all become more or less familiar, but it is not often that the ravages which Yellow Fever has made upon the Mississippi delta the last year, occur in the experience of a generation. Nor are such thoughts as the following, from the pen of the distinguished Dowler, often noticed, after an epidemic of the kind has subsided:

"*Yellow Fever in New Orleans—Beautiful and Horrid Pictures.*"

"*Contrasts.*—The earth, air and sky seemed to be in the midst of the pestilence; such as Goethe described, which appear in the strongest contrast, when humanity is desolated:

"Know'st thou the land where the pale citron grows,
And the gold orange through dark foliage glows?
A soft wind flutters through the deep blue sky,
The myrtle blooms, and towers the laurel high.
Know'st thou it well?
"O there with thee!
O that I might, my own beloved one, flee!"

Yet, in the midst of such scenes, the Angel of Death poured out the phials of his wrath. Coffin rumbled after coffin; the funeral columns defiled almost constantly for months from every street

"To join
The innumerable caravan that moves
To the pale realms of shade."

" *Sun-set.*—As the day declined the funeral march became dense, continuous and often blended. It was then that nature was serenest, while the sun was sinking into the cypress forests, his slantest rays dying with variegated hues, the trembling waves of the river, recalling to the mind the sublime descriptions of Scott and Goethe; the first relating to a tropical sun-set, and the second one in the temperate zone:

" No pale gradations quench his ray,
No twilight dews his wrath allay;
With disk like battle target red,
He rushes to his burning bed,
Dyes the wide wave with bloody light,
Then sinks at once—and all is night."

See how the green-girt cottages shimmer in the setting sun! He bends and sinks. Yonder he hurries off and quickens other life. Oh! that I have no wing to lift me from the ground, to struggle after, forever after him! I should see, in everlasting evening beams, the stilly worl at my feet—every hight on fire—every vale in repose—the silver brook flowing into golden streams. I hurry on to drink his everlasting light—the day before me and the night behind—the heavens above and under me the waves."

"*Poetry of the Pestilence.*—These contrasts between the beauty and repose of nature, and the march of death, gave rise to several poetical contributions, which were cut short in some cases by " the pest king," whose power they were recording—the muse trailing her fast-failing wings in the polluted blood and black vomit:

—————————" All hoping is past!
The black draught of death is ejected at last!"

So reads one the unfinished black vomit poems of 200 lines, by a physician who died of the *vomito*, which he sung.

Another says:

" The sun sinks down o'er each death-crowded street,
Whilst dread, delirious screams the hearing greet;
Night settles o'er with awe and fear and gloom.
What means yon glaring blaze, yon cannon's boom?
Ha! victory's tokens for the Conqueror Death!
Who slays his thousand by the Fever's breath!"

" *Night.*—Night was ushered in, for a short period with cannonading, and for a considerable time with conflagrations from burning tar, the towering flames of which cast a sickly flickering light among the streets, upon the river, and into many a dilapidated window, upon yellow rigid corpses, awaiting interment on the morrow."—*Dr. Dowling.*

